# Implications of asymptomatic carriers for infectious disease transmission and control

**DOI:** 10.1098/rsos.172341

**Published:** 2018-02-14

**Authors:** Rebecca H. Chisholm, Patricia T. Campbell, Yue Wu, Steven Y. C. Tong, Jodie McVernon, Nicholas Geard

**Affiliations:** 1 Centre for Epidemiology and Biostatistics, Melbourne School of Population and Global Health, The University of Melbourne, Melbourne, Victoria, Australia; 2 School of Computing and Information Systems, Melbourne School of Engineering, The University of Melbourne, Melbourne, Victoria, Australia; 3 Modelling and Simulation Research Group, Murdoch Childrens Research Institute, Royal Children’s Hospital, Parkville, Victoria, Australia; 4 The Peter Doherty Institute for Infection and Immunity, The University of Melbourne and Royal Melbourne Hospital, Melbourne, Victoria, Australia; 5 Wesfarmers Centre of Vaccines & Infectious Diseases, Telethon Kids Institute, University of Western Australia, Subiaco, Western Australia, Australia; 6 Victorian Infectious Diseases Service, The Royal Melbourne Hospital, and the University of Melbourne, The Peter Doherty Institute for Infection and Immunity, Melbourne, Victoria, Australia; 7 Global and Tropical Health Division, Menzies School of Health Research, Charles Darwin University, Darwin, Northern Territory, Australia

**Keywords:** pathogen carriage, asymptomatic infection, mathematical modelling, epidemiology, infectious disease control

## Abstract

For infectious pathogens such as *Staphylococcus aureus* and *Streptococcus pneumoniae*, some hosts may carry the pathogen and transmit it to others, yet display no symptoms themselves. These asymptomatic carriers contribute to the spread of disease but go largely undetected and can therefore undermine efforts to control transmission. Understanding the natural history of carriage and its relationship to disease is important for the design of effective interventions to control transmission. Mathematical models of infectious diseases are frequently used to inform decisions about control and should therefore accurately capture the role played by asymptomatic carriers. In practice, incorporating asymptomatic carriers into models is challenging due to the sparsity of direct evidence. This absence of data leads to uncertainty in estimates of model parameters and, more fundamentally, in the selection of an appropriate model structure. To assess the implications of this uncertainty, we systematically reviewed published models of carriage and propose a new model of disease transmission with asymptomatic carriage. Analysis of our model shows how different assumptions about the role of asymptomatic carriers can lead to different conclusions about the transmission and control of disease. Critically, selecting an inappropriate model structure, even when parameters are correctly estimated, may lead to over- or under-estimates of intervention effectiveness. Our results provide a more complete understanding of the role of asymptomatic carriers in transmission and highlight the importance of accurately incorporating carriers into models used to make decisions about disease control.

## 1. Introduction

For many infectious diseases, an unknown fraction of infected hosts are able to spread disease while remaining symptom-free. We designate these asymptomatic hosts, ‘asymptomatic carriers’ or ‘carriers’ and reserve the term ‘symptomatic infectious’ for those with observable clinical manifestation, who are more readily identified and targeted by disease control efforts.

The elusive nature of asymptomatic carriers has three important implications for understanding and controlling infectious diseases with carriers. First, incidence data typically only reflect symptomatic cases of infection, making the true extent of asymptomatic carriage for particular diseases difficult to assess. For example, estimates of the population-level prevalence of influenza carriage range from 5 to 35% [1]. For Ebola virus, 27–71% of all infections are estimated to be asymptomatic [2,3]. Similarly, estimates of the population-level prevalence of meningococcus carriage can approach 100% in closed and semi-closed populations, but are in the range of 10–35% in young adults [4].

Second, the sparsity of direct evidence of asymptomatic carriers makes interpretation of the epidemiological record difficult, creating uncertainty when defining details of the carrier state and a pathogen’s more complex natural history of disease [5,6]. For example, estimating the duration of asymptomatic carriage is difficult due to limited data on time of infection and of subsequent pathogen clearance [7].

Third, the presence of asymptomatic carriers undermines control interventions that rely on identifying infectious cases, such as border monitoring or isolating and/or treating infectious cases [8]. Asymptomatic carriers can also affect the efficiency of interventions targeting susceptible individuals, such as vaccination or widespread prophylaxis, because susceptible individuals can be difficult to distinguish from asymptomatic carriers [9]. One serious implication of unknowingly treating carriers is that it exerts selective pressure that may contribute to the emergence and transmission of drug-resistant strains [10,11].

Mathematical modelling has helped to unravel some of the complexities of asymptomatic carriage and its implications for control [9,12–14]. For example, modelling studies of influenza [13] and community-associated methicillin-resistant *Staphylococcus aureus* (MRSA) [14] found that control strategies targeting symptomatically infected hosts but not asymptomatic carriers are actually incapable of achieving disease control, even when multiple control types are implemented. Modelling has also revealed the importance of accounting for asymptomatic carriers in epidemiological models. For example, under certain conditions, neglecting pre-symptomatic influenza transmission was shown to overestimate the impact of interventions targeting symptomatically infected hosts [12]. Neglecting asymptomatic carriers in a model of Ebola virus transmission was shown to significantly overestimate the projected cumulative incidence of symptomatic infections, as well as overestimate the population-level vaccination coverage needed to contain epidemics [9]. Accordingly, if carriage is not appropriately represented in mathematical models, there is a risk that they may incorrectly assess the viability of an intervention, potentially leading to missed opportunities for controlling disease or, conversely, the implementation of an ineffective intervention. Therefore, without sufficient knowledge of asymptomatic carriers the parametrization and validation of mathematical models is compromised. More fundamentally, this uncertainty also undermines the choice of model used to represent the transmission and natural history of an infectious disease, as evidenced by the variation in model structure observed across mathematical modelling studies of MRSA [14–22]. This uncertainty in the underlying structure of mathematical models of infectious diseases with asymptomatic carriers casts further doubt on evaluations of interventions from mathematical models, potentially hindering the decision-making process of policy-makers.

There is a need to understand how the structure of a mathematical model, specifically, how a model depicts asymptomatic carriage, affects its behaviour. To address this need, here we review existing mathematical models of infectious disease transmission for the purpose of identifying the types of model structures that are used to describe transmission with asymptomatic carriers. Based on this review, we define a new model of pathogen transmission that enables us to systematically compare the behaviour of alternative model structures and how they influence the estimated impact of control interventions. First, we analyse this model to establish the contribution of asymptomatic carriers to measures of pathogen transmission, the prevalence of symptomatic infections and the total infection (both symptomatic and asymptomatic) prevalence. We then consider how excluding asymptomatic carriers from this model could lead to the misinterpretation of data and the conditions under which this obscures the most important drivers of pathogen transmission. Finally, we evaluate the impact of a range of interventions on transmission and the prevalence of disease, and discuss how the choice of model structure might affect the assessment of these interventions.

Our analysis reveals that interventions that alter the relative incidence of symptomatic infections compared to asymptomatic carriers are particularly vulnerable to being incorrectly assessed by models with inappropriate structure. Examples of this type of intervention are the multivalent *Streptococcus pneumoniae* vaccines. These vaccines only protect against about 10% of this pathogen’s 90 identified serotypes. Those included in the vaccine are selected for their predominance as causes of invasive pneumococcal disease, allowing less virulent (carriage) strains to persist [23], essentially reducing the relative incidence of symptomatic infections versus asymptomatic carriage. Our study provides a better understanding of the dynamic behaviour of models that include asymptomatic carriers, will inform more appropriate design of future models and contribute to a more complete understanding of the impact of asymptomatic carriage on pathogen dynamics.

## 2. Material and methods

### 2.1. Literature review and model categorization

The literature review identified 132 articles presenting models of pathogen transmission accounting for asymptomatic carriers, of which 42 articles (presenting a total of 45 models) met our inclusion criteria (for more details see electronic supplementary material, appendix A). Pathogens modelled in these studies include *S. aureus* (nine studies), hepatitis B virus (4), *Neisseria gonorrhoeae* (3), *Chlamydia trachomatis* (2), *Neisseria meningitidis* (2), hand-foot-mouth disease enterovirus (2), *Mycoplasma mycoides* subsp. mycoides small colony (2), *S. pneumoniae* (1), norovirus (1), *Trichomonas vaginalis* (1), rotavirus (1), *Salmonella enterica* serotype Typhi (1), *Bordetella pertussis* (1), *Acinetobacter baumannii* (1), Middle East respiratory syndrome coronavirus (1), A/H1N1 pandemic influenza virus (1) and HIV (1), while eight studies considered unidentified abstract pathogens.

To make comparisons between these models, we collapsed each model to a generic form where hosts belong to one of four states: susceptible (*S*); asymptomatic carriers (*C*); symptomatically infectious (*I*); and immune (*R*). Models with an exposed state (*E*) were collapsed in a way such that any exposed and adjacent infectious states (either *C* or *I*) were combined into single infectious states. For example, a pathway S→E→C collapsed into the form S→C and S→E→I into S→I. We identified 23 generic models that differed according to flows representing transmission events (leading to asymptomatic infection: S→C, or symptomatic infection: S→I), flows representing a change in infection state (disease regression: I→C, disease progression: C→I), and flows corresponding to clearance of the pathogen (from asymptomatic infection: C→S or *R*, from symptomatic infection: I→S or *R*). These generic models were sorted according to whether or not hosts can experience infection more than once (because infection leads to either waning or zero immunity to reinfection, or either lifelong immunity or death), and then whether disease progression (C→I) and/or disease regression (I→C) are allowed (for more details see electronic supplementary material, figure S1).

The transmission dynamics of pathogens that can cause infection more than once in individual hosts can differ significantly from those that cannot reinfect hosts following clearance of an infection [24]. This is likely to also hold true when infectious cases can be either asymptomatic or symptomatic. Therefore, we disregard models that do not allow reinfection. This leaves 15 model types in our analysis out of the original 23 found in the literature review.

The remaining models include those with and without the temporary immune class *R* and describe the transmission of either *S. aureus*, *N. gonorrhoeae*, *C. trachomatis*, *N. meningitidis*, hand-foot-mouth disease enterovirus, *S. pneumoniae*, norovirus, *T. vaginalis*, rotavirus, *S. enterica* serotype Typhi, *B. pertussis*, *A. baumannii* or unidentified abstract pathogens. To make comparisons between these models, we collapsed those with the respective flows C→R→S and I→R→S into models with respective flows C→S and I→S, which reduced the total number of models remaining in our analysis from 15 to 10. Such a simplification does not alter model behaviour at long time scales and measures of disease persistence in simple models without carriers [25] (because it is essentially removing a delay in the infection pathway and changing neither the infectious periods of hosts nor the transmission rates), and we expect this simplification to have a similarly mild effect on dynamics in simple models with carriers.

**Figure 1.**
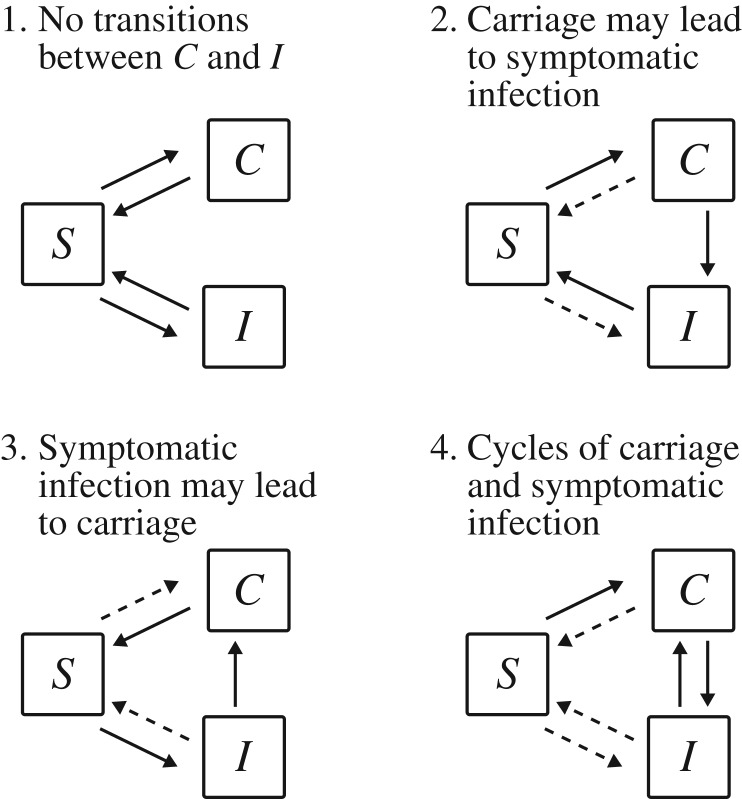
Categorization of mathematical epidemiological models used to study infectious disease transmission when there are asymptomatic carriers (a list of the associated references is provided in electronic supplementary material, figure S1). Here, *C* indicates asymptomatic carriers, *I* symptomatic infectives and *S* susceptible hosts, while solid arrows indicate state transitions that are common to all models in a category, and broken arrows indicate transitions that are present in at least one but not all models in a category.

**Figure 2.**
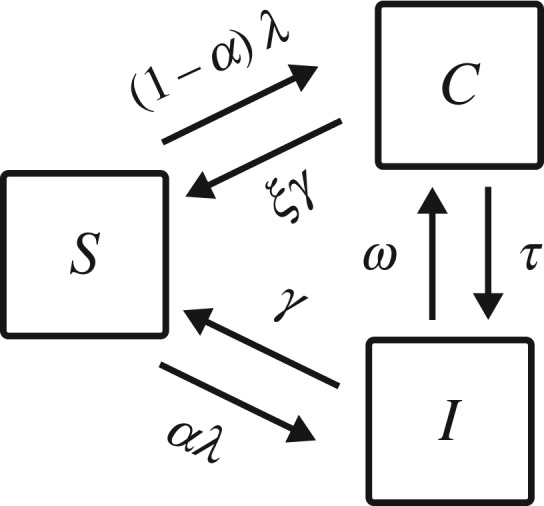
Flow diagram illustrating the structure of the model describing the transmission of a pathogen with both symptomatic and asymptomatic infection in its life cycle. *S*, susceptible hosts; *C*, asymptomatic carriers; *I*, symptomatic infectives; *λ*(*C*,*I*)=*β*(*ηC*+*I*)/*N*, force of infection; *α*, proportion of new cases that are symptomatic; *ξγ*, clearance rate of asymptomatic carriage; *γ*, clearance rate of symptomatic infections; *τ*, rate of progression to symptomatic infection; *ω*, rate of regression to carriage.

We categorized the remaining 10 generic models according to the presence or absence of flows between the asymptomatic carriage state *C* and symptomatic infection state *I* such that each category contains models where either (1) no transitions exist between *C* and *I*; (2)–(3) transitions can occur in one direction only; and (4) transitions can occur in both directions, as shown in figure 1. Each of these broad model categories represent special cases of the model of transmission with asymptomatic carriage shown in figure 2 and described in the next section.

### 2.2. A model of transmission with asymptomatic carriage

We consider the following model of transmission of a non-immunizing infectious disease with asymptomatic carriage:

2.1 $$\begin{array}{rl} \frac{dC}{dt} & =(1-\alpha )\lambda (C,I)S-(\xi \gamma +\tau )C+\omega I\\ \;\text{and}\;\frac{dI}{dt} & =\alpha \lambda (C,I)S+\tau C-(\gamma +\omega )I, \end{array}\}$$

with *S*=*N*−*C*−*I* (shown schematically in figure 2). In this model, there are three host states: susceptible, asymptomatic carrier and symptomatic infected with respective population sizes *S*≥0, *C*≥0 and *I*≥0. The force of infection is given by *λ*(*C*,*I*)=*β*(*ηC*+*I*)/*N*, where *β*>0 is the pathogen’s transmission rate during symptomatic infection, *η*>0 is the relative transmission rate of asymptomatic carriers compared to symptomatically infected hosts, and *N*>0 is the total host population size (which is assumed to be constant).

The model represents the dynamics of a pathogen where the time scale of disease is typically much faster than the time scale of host births and deaths, and where infection typically does not kill hosts, such as norovirus, an influenza virus or hand-foot-mouth disease. Hence, the demographic effects of the population are ignored in the model and there is no input or output from the host population. However, in electronic supplementary material, appendix C, we provide details of an equivalent model accounting for host demography that would better describe pathogens with longer time scales of disease.

New cases of infection are either symptomatic or asymptomatic with complementary proportions *α* and 1−*α*. Hosts may also regress to asymptomatic carriage from symptomatic infection or progress to symptomatic infection from asymptomatic carriage at respective *per capita* rates *ω*≥0 and *τ*≥0. The model allows natural recovery to occur from both symptomatic and asymptomatic infection, resulting in hosts returning to the susceptible class at respective *per capita* rates *γ*≥0 and *ξγ*≥0, where *ξ*≥0 is the relative recovery rate of asymptomatic carriers compared to symptomatic infectives. Hence, neither recovery from infection nor recovery from carriage confers protective immunity. A summary of these parameters and variables is provided in table 1.

**Table 1.** Parameter and variable definitions for the model of infectious disease transmission in the presence of asymptomatic carriers.

parameter	description
*I*	number of symptomatically infected hosts
*C*	number of asymptomatic carriers
*S*	number of susceptible hosts
s *N*	total number of hosts
*β*	transmission rate of pathogen from symptomatically infected host
*η*	relative infectiousness of carriers compared to symptomatically infected hosts
*α*	proportion of new infections that are symptomatic
*γ*	clearance rate of symptomatic infections
*ξ*	relative clearance rate of carriage compared to symptomatic infection
*τ*	rate of progression to symptomatic infection
*ω*	rate of regression to carriage
$R_{0}$	reproduction number
$R_{C}$	host-specific reproduction number for asymptomatic carriers
$R_{I}$	host-specific reproduction number for symptomatic infections
A	relative reproduction potential of asymptomatic carriers
P	relative endemic prevalence of asymptomatic carriers versus symptomatic infections

In this framework, the four model categories identified in the literature review (figure 1) are defined according to whether hosts can transition between the asymptomatic carriage and symptomatic infection states during one case of infection, i.e. whether their progression and regression rates *τ* and *ω* are non-zero. Specifically, models in category 1 are characterized by *τ*=*ω*=0, category 2 by *τ*>0 and *ω*=0, category 3 by *τ*=0 and *ω*>0, and category 4 by *τ*>0 and *ω*>0.

## 3. Results

### 3.1. Asymptomatic carriers can be key drivers of pathogen transmission

#### 3.1.1. The basic reproduction number

The basic reproduction number $R_{0}$ is the average number of secondary cases of infection caused by a single case of infection in an entirely susceptible population. For the model defined by the system of equations (2.1),

3.1 $$R_{0}=(1-\alpha )R_{C}+\alpha R_{I},$$

where $R_{C}$ is the host-specific reproduction number of asymptomatic carriers and $R_{I}$ is the host-specific reproduction number of symptomatic infectives such that

3.2 $$\begin{array}{rl} R_{C} & =\frac{\eta \beta }{\xi \gamma +\tau (\gamma /(\omega +\gamma ))}+\left(\frac{\tau }{\tau +\xi \gamma }\right)\times \frac{\beta }{\gamma +\omega (\xi \gamma /(\tau +\xi \gamma ))}\\ \;\text{and}\;R_{I} & =\frac{\beta }{\gamma +\omega (\xi \gamma /(\tau +\xi \gamma ))}+\left(\frac{\omega }{\omega +\gamma }\right)\times \frac{\eta \beta }{\xi \gamma +\tau (\gamma /(\omega +\gamma ))}. \end{array}\}$$

$R_{C}$ is heuristically derived as follows. Following the method outlined in [26], a fraction *h*_1_=*τ*/(*τ*+*ξγ*) of asymptomatic carriers progress to symptomatic infection, and a fraction *h*_2_=*ω*/(*ω*+*γ*) of symptomatic infections regress to asymptomatic carriage. Hence, a fraction *h*_1_ of newly infected asymptomatic carriers progress to symptomatic infection at least once, a fraction $h_{1}^{2}h_{2}$ progress to symptomatic infection at least twice and a fraction $h_{1}^{k}h_{2}^{k-1}$ of newly infected asymptomatic carriers progress to symptomatic infection at least *k* times, spending an average of *g*_1_=1/(*ω*+*γ*) time units with symptoms per instance of symptomatic infection. Similarly, all newly infected asymptomatic carriers experience asymptomatic infection at least once, a fraction *h*_1_*h*_2_ experience asymptomatic infection at least twice and a fraction $h_{1}^{k}h_{2}^{k}$ of newly infected asymptomatic carriers experience asymptomatic infection at least *k* times, spending an average of *g*_2_=1/(*τ*+*ξγ*) time units without symptoms during each instance of asymptomatic carriage. Thus a newly infected asymptomatic carrier spends on average $g_{1}(h_{1}+h_{1}^{2}h_{2}+\cdots )=g_{1}h_{1}/(1-h_{1}h_{2})=\tau /((\tau +\xi \gamma )(\gamma +\gamma \omega \xi /(\tau +\xi \gamma )))$ time units with symptomatic infection, and $g_{2}(1+h_{1}h_{2}+h_{1}^{2}h_{2}^{2}+\cdots )=g_{2}/(1-h_{1}h_{2})=1/(\gamma \xi +\gamma \tau /(\omega +\gamma ))$ time units with asymptomatic carriage over its expected lifetime. Multiplying these two terms by the respective transmission rates *β* (from symptomatic infections) and *ηβ* (from asymptomatic carriers), and summing gives $R_{C}$. A similar argument can be made to heuristically derive $R_{I}$ (for the formal derivation of $R_{0}$ see electronic supplementary material, appendix B).

If asymptomatic carriers and symptomatically infected hosts experience infection at a proportionally consistent rate so that *ξ*=*η*, then the host-specific reproduction numbers are identical and equal to the population-level basic reproduction number $R_{0}$ so that $R_{C}=R_{I}=R_{0}=\beta /\gamma .$ Furthermore, if a pathogen only causes either asymptomatic carriage or symptomatic infection, then the host-specific reproduction number of asymptomatic carriers $R_{C}$ or symptomatic infectives $R_{I}$ is equivalent to the basic reproduction number $R_{0}$.

The basic reproduction number $R_{0}$ determines the long-time behaviour of this system, that is, whether the pathogen will become extinct in the host population or endemic [26]. In electronic supplementary material, appendix B, we show that $R_{0}$ is a threshold for global stability of the *disease-free equilibrium* (*C*=*I*=0), which is stable when $R_{0}<1$; when $R_{0}>1$, the *endemic equilibrium* ($C=\hat{C}>0$, $I=\hat{I}>0$) is biologically meaningful and stable.

For the analysis that follows, it is convenient to define the *relative reproduction potential of asymptomatic carriers* A, where

3.3 $$A=\frac{\eta }{\xi }.$$

This compound parameter is the relative host-specific reproduction number of asymptomatic carriers $R_{C}$ compared to symptomatic hosts $R_{I}$ in the absence of the other host type. It is also a measure of the relative reproduction number of carriers when the other host type is present as $R_{C}>R_{I}$ if and only if A>1.

The relative reproduction potential of asymptomatic carriers A is always positive and finite in our model when recovery from both asymptomatic carriage and symptomatic infection is possible (so that *γ*>0 and *ξ*>0). If recovery is only possible from symptomatic infection (so that *ξ*=0), then A→∞. Conversely, if recovery is only possible from asymptomatic carriage (so that *γ*=0 and ξ→∞ in a way such that *γξ*→a constant), then A→0.

#### 3.1.2. The prevalence of infection

The total prevalence of infection *P*(*t*) is the proportion of cases in a host population at a given time *t* accounting for both asymptomatic carriers and symptomatically infected cases. In our model *P*(*t*) is given by

3.4 $$P(t)=P_{C}(t)+P_{I}(t),$$

where *P*_C_(*t*) is the prevalence of asymptomatic carriers and *P*_I_(*t*) is the prevalence of symptomatic infection at time *t* such that

$$P_{C}(t)=\frac{C(t)}{N}\;\text{and}\;P_{I}(t)=\frac{I(t)}{N}.$$

At the endemic equilibrium, the respective infection-type prevalences are

$${\hat{P}}_{C}=\lim_{t\to \infty }P_{C}(t)\;\text{and}\;{\hat{P}}_{I}=\lim_{t\to \infty }P_{I}(t).$$

These quantities can be calculated by setting the system of equations (2.1) to zero (as shown in electronic supplementary material, appendix B) so that

3.5 $$0=(1-\alpha )\lambda (\hat{C},\hat{I})\hat{S}-\;(\xi \gamma +\tau )\hat{C}+\omega \hat{I}\;\text{and}\;0=\alpha \lambda (\hat{C},\hat{I})\hat{S}+\tau \hat{C}-(\gamma +\omega )\hat{I}.$$

By adding equations (3.5) together and rearranging, we see that $\lambda (\hat{C},\hat{I})\hat{S}=\gamma (\hat{I}+\xi \hat{C})$ which, when substituted back into either of the equations in (3.5), reveals the following expression for the relative endemic prevalence P of asymptomatic carriers to symptomatic infections:

3.6 $$P=\frac{{\hat{P}}_{C}}{{\hat{P}}_{I}}=\frac{\omega +\gamma (1-\alpha )}{\tau +\xi \gamma \alpha }\geq 0.$$

When P>1, asymptomatic carrier prevalence will outweigh the prevalence of symptomatically infected cases; otherwise symptomatic infections will be the most prevalent. Loosely speaking, the quantity P can also be interpreted as the ratio of the net per capita rates of transitioning from *I*→*C* (both directly and indirectly via *S*), and from *C*→*I* (both directly and indirectly via *S*). In electronic supplementary material, appendix B, we show that P is related to the basic reproduction number $R_{0}$ according to

3.7 $$R_{0}=\frac{\beta }{\gamma }\left(\frac{1+\xi PA}{1+\xi P}\right),$$

as well as the respective infection-type prevalences ${\hat{P}}_{C}(t)$ and ${\hat{P}}_{I}(t)$ so that

3.8 $${\hat{P}}_{C}=\frac{P}{1+P}\left(1-\frac{1}{R_{0}}\right)\;\text{and}\;{\hat{P}}_{I}=\frac{1}{1+P}\left(1-\frac{1}{R_{0}}\right).$$

Finally, the total endemic infection prevalence is

3.9 $$\hat{P}=\lim_{t\to \infty }P(t)=1-\frac{1}{R_{0}}.$$

Clearly, the endemic prevalence is an increasing function of the basic reproduction number $R_{0}$ and is only positive when $R_{0}>1$.

### 3.2. Excluding asymptomatic carriers from mathematical models can obscure the key drivers of pathogen transmission

When confronted with a sparsity of direct evidence of asymptomatic carriage, it may be tempting to assume asymptomatic carriers play a minimal role in pathogen transmission and to disregard them in transmission models. Here, we consider the consequences of such an assumption for estimating transmission from symptomatic infections and of a disease’s basic reproduction number and prevalence.

Excluding asymptomatic carriers from our model transforms it into a standard susceptible-infected-susceptible (SIS) model. In the SIS model, the basic reproduction number $R_{0,\mathrm{SIS}}$ has the standard form $R_{0,\mathrm{SIS}}=\beta_{\mathrm{SIS}}/\gamma_{\mathrm{SIS}}$, which is simply the ratio of transmission rate to recovery rate. The dynamics of the SIS model are well known [27] and are determined by the parameters defining $R_{0,\mathrm{SIS}}$, i.e. *β*_SIS_ and *γ*_SIS_. Similar to our model, in this SIS model the pathogen will become extinct if $R_{0,\mathrm{SIS}}\leq 1$. Otherwise, the pathogen will become endemic with total infection prevalence ${\hat{P}}_{\mathrm{SIS}}=1-1/R_{0,\mathrm{SIS}}$.

If the basic reproduction number can be calculated independently of the model (i.e. from individual-level contact tracing data at the start of an epidemic), then the basic reproduction number will be equivalent in both models so that

3.10 $$R_{0,\mathrm{SIS}}=R_{0}.$$

The respective rates *γ*_SIS_ and *γ* of recovery from symptomatic infection in both models should also be equivalent so that

3.11 $$\gamma_{\mathrm{SIS}}=\gamma$$

since they are simply the inverse of the duration of symptomatic infection. When these two sets of parameters are known, the associated models can be used to calculate the respective rates *β*_SIS_ and *β* of transmission from symptomatically infected hosts. Hence, these rates may vary between the two models. By combining equations (3.7) and (3.10)–(3.11), we see that the ratio of these transmission rates satisfies

3.12 $$\frac{\beta_{\mathrm{SIS}}}{\beta }=\frac{1+AP\xi }{1+P\xi }.$$

Clearly, *β*_SIS_>*β* when A>1, *β*_SIS_<*β* when A<1 and *β*_SIS_=*β* when A=1. Therefore, given empirically observed values for the basic reproduction number and recovery rate from symptomatic infection, fitting an SIS model will overestimate the transmission rate from symptomatically infected hosts when asymptomatic carriers have a higher reproduction potential than symptomatically infected hosts. Otherwise, the SIS model underestimates the transmission rate from symptomatically infected hosts.

Alternatively, if it is not possible to estimate the basic reproduction number independently, it can be estimated using the respective models. In this case, and given empirically observed values for the recovery rate from symptomatic infection and the transmission rate from symptomatically infected hosts, each model may provide different estimates of the basic reproduction number. Combining equations (3.7) and (3.11), we see that the basic reproduction number of our model $R_{0}$ relates to that of the SIS model $R_{0,\mathrm{SIS}}$ according to the equation

3.13 $$R_{0}=R_{0,\mathrm{SIS}}\left(\frac{1+AP\xi }{1+P\xi }\right).$$

Clearly, $R_{0}>R_{0,\mathrm{SIS}}$ when A>1, $R_{0}<R_{0,\mathrm{SIS}}$ when A<1 and $R_{0}=R_{0,\mathrm{SIS}}$ when A=1. We can also deduce that $\hat{P}>{\hat{P}}_{\mathrm{SIS}}$ when A>1, $\hat{P}<{\hat{P}}_{\mathrm{SIS}}$ when A<1 and $\hat{P}={\hat{P}}_{\mathrm{SIS}}$ when A=1 because $\hat{P}$ and ${\hat{P}}_{\mathrm{SIS}}$ are the same increasing functions of $R_{0}$ and $R_{0,\mathrm{SIS}}$, respectively. We also show that the condition ${\hat{P}}_{I}<{\hat{P}}_{\mathrm{SIS}}$ always holds true when A≤1, and can also hold when A>1 if P is large enough. Otherwise, ${\hat{P}}_{I}>{\hat{P}}_{\mathrm{SIS}}$ (for details see electronic supplementary material, appendix D).

Therefore, when the recovery rates and the transmission rates are held fixed between the two models, the SIS model overestimates the basic reproduction number, the total endemic prevalence and the endemic prevalence of disease when asymptomatic carriers have a lower reproduction potential than symptomatically infected hosts. Otherwise, the SIS model underestimates the basic reproduction number, the total endemic prevalence and possibly the endemic prevalence of disease if symptomatic infections appear at a sufficiently fast rate.

To explain this set of results, we note that in our model, asymptomatic carriers arise from the same pool of susceptible hosts as symptomatic infectives so that if an asymptomatic carrier exists, it is at the cost of a symptomatic case. If asymptomatic carriers then have a lower reproduction potential than symptomatic infectives, their presence detracts from the overall transmission and reproduction potential of the pathogen due to lost opportunities for more productive symptomatic cases. When the basic reproduction number is fixed between the two models, the extra contribution to transmission from symptomatically infected hosts required for the overall reproduction number to match that calculated from the epidemiological data is only apparent in our model; the SIS model underestimates transmission from symptomatically infected hosts. On the other hand, if asymptomatic carriers have a higher reproduction potential than symptomatic infectives, they will increase overall transmission and infection reproduction. In this case, and when the basic reproduction number is fixed between the two models, the SIS model overestimates the contribution to transmission by symptomatically infected hosts.

**Figure 3.**
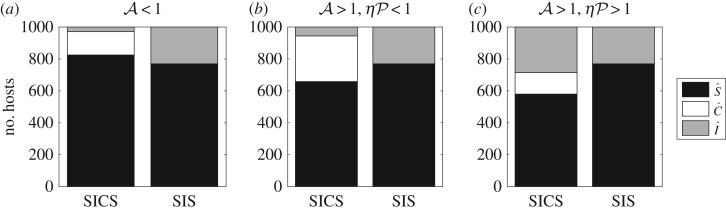
Comparisons between the numbers of individuals in the different host compartments at endemic equilibrium ($\hat{S}$ shown in black, $\hat{C}$ shown in white and $\hat{I}$ shown in grey) in the full model (SICS model) versus the SIS model when the transmission rates and recovery rates from symptomatic infection are fixed between the two models. (*a*) The overall endemic prevalence is greater in the SIS model ($\hat{P}<{\hat{P}}_{\mathrm{SIS}}$, A<1); (*b*,*c*) the overall endemic prevalence is greater in the SICS model ($\hat{P}>{\hat{P}}_{\mathrm{SIS}}$, A>1). In (*b*), the predicted number of symptomatic cases is greater in the SIS model (${\hat{P}}_{I}<{\hat{P}}_{\mathrm{SIS}}$, ηP<1), whereas in (*c*) the predicted number of symptomatic cases is greater in the SICS model (${\hat{P}}_{I}>{\hat{P}}_{\mathrm{SIS}}$, ηP>1). Parameter values are provided in electronic supplementary material, appendix E.

We illustrate these results in figure 3, where the sizes of the endemic subpopulations $\hat{S}$, $\hat{C}$ and $\hat{I}$ in our model are compared to those of the SIS model when the transmission and recovery rates from symptomatic infection are fixed between the two models. The size of the susceptible pool is noticeably smaller in the SIS model when A<1 (figure 3*a*), and larger if this is not the case (figure 3*b*,*c*). Even when less prevalent, asymptomatic carriers can be responsible for the majority of transmissions when P<1<ηP, as shown in figure 3*c*. A necessary but not sufficient condition for this to occur is that asymptomatic carriers are more infectious than symptomatic hosts so that *η*>1 (see electronic supplementary material, appendix D).

### 3.3. Imperfect knowledge of asymptomatic carriage can result in misleading assessments of strategies for control

Now we assess how control interventions that target different aspects of the disease process may affect the basic reproduction number $R_{0}$, the total endemic prevalence $\hat{P}$ and the endemic prevalence of symptomatic infection ${\hat{P}}_{I}$. Each intervention is modelled by a change in one parameter of the model. For example, a vaccine that acts to decrease the susceptibility of hosts to both symptomatic infection and asymptomatic carriage is modelled as a decrease in the transmission rate *β*. We also consider interventions that change *η* (e.g. by quarantining symptomatic infectives), *γ* (e.g. through a mass drug administration), *ξ* (e.g. by treating only symptomatic cases), *ω* (e.g. by providing treatment that targets symptoms, not pathogen clearance), *τ* (e.g. by improving sanitation) and *α* (e.g. through a vaccine targeting pathogen strains associated with symptomatic infection, allowing carriage strains to persist).

First, we determine whether the slope of $R_{0}$ (and thus the slope of $\hat{P}$ because it is an increasing function of $R_{0}$) in the direction of any of these model parameters switches from being positive to negative as other parameters in the model are changed. If a switch in the slope of $R_{0}$ in the direction of one of these parameters is possible, then there is a risk that the corresponding intervention will be predicted to either reduce or increase transmission but lead to the opposite effect if estimates of other model parameters are inaccurate.

It is straightforward to show that the condition

3.14 $$\frac{\partial R_{0}}{\partial x}>0,$$

always holds true for the parameters *x*∈{*η*,*β*}, and never holds true for the parameters *x*∈{*γ*,*ξ*}, which makes intuitive sense because increasing the infectivity of either infection type (through increasing *β* or *η*) or prolonging their periods of infectiousness (through decreasing *γ* or *ξ*) will always result in more secondary infections on average per infectious case. For parameters controlling the relative frequency of appearance of asymptomatic carriers (*x*∈{*ω*,*τ*,*α*}), the reproduction potential of carriers A determines whether or not condition (3.14) holds true. For *x*∈{*ω*}, condition (3.14) only holds true when A>1. If A<1, then condition (3.14) holds true for *x*∈{*τ*,*α*}. In electronic supplementary material, figure S2, we illustrate the dependence of $\partial R_{0}/\partial \tau$ on A. Clearly, decreasing *τ* causes $R_{0}$ to increase only if A<1; otherwise it decreases.

We also considered the effects of the same set of interventions on the endemic prevalence of disease ${\hat{P}}_{I}$. For *x*∈{*β*,*η*}, a similar result is obtained: $\partial {\hat{P}}_{I}/\partial x>0$ always holds true for *x*∈{*β*,*η*}. For parameters controlling the relative frequency of appearance of asymptomatic carriers (*x*∈{*ω*,*τ*,*α*}), the reproduction potential again plays a key role in determining whether $\partial {\hat{P}}_{I}/\partial x>0$ holds true. When A<1, decreasing the relative frequency of appearance of symptomatic infectives (by decreasing *τ* or *α*, or increasing *ω*) will always result in a lower endemic prevalence of disease ${\hat{P}}_{I}$. If, on the other hand, A>1, then decreasing the relative frequency of appearance of symptomatic infectives may actually increase ${\hat{P}}_{I}$ (for details see electronic supplementary material, appendix D and figure S4).

Overall, our analyses highlight how the relative reproduction potential of carriers A largely determines the effects of interventions on a pathogen’s basic reproduction number $R_{0}$, total endemic prevalence $\hat{P}$ and the endemic prevalence of disease ${\hat{P}}_{I}$, when interventions are designed to alter the relative frequency of appearance of asymptomatic carriers (i.e. change *τ*, *ω* and *α*).

**Figure 4.**
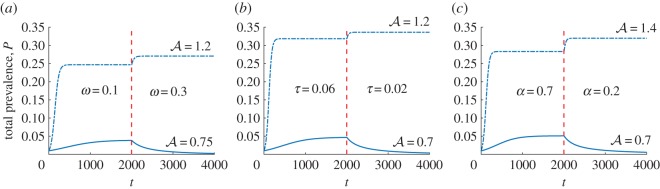
The total prevalence *P* calculated using the model accounting for asymptomatic carriers is shown as a function of time *t* before and after an intervention administered at time *t*=2000 (indicated by the dashed red line) for two different values of the relative reproduction potential of carriers A that are greater than unity (dashed-dot line) and less than unity (solid line). Here, the intervention (*a*) increases the regression rate *ω* from *ω*=0.1 to *ω*=0.3; (*b*) reduces the progression rate *τ* from *τ*=0.06 to *τ*=0.02; (*c*) reduces the proportion *α* of new infections that are symptomatic from *α*=0.7 to *α*=0.2. Parameter values are provided in electronic supplementary material, appendix E.

In figure 4, we provide examples of how A affects the outcomes of interventions targeting either the regression rate *ω* (figure 4*a*), the progression rate *τ* (figure 4*b*) or the symptomatic proportion *α* (figure 4*c*). The corresponding endemic prevalences of asymptomatic carriers, symptomatic infections and total prevalence before and after these interventions are shown in electronic supplementary material, figure S3. Accordingly, if estimates of A are inaccurate there is the potential for an effective intervention to be assessed as unviable by the model, or for an ineffective intervention to be recommended for implementation.

## 4. Discussion

The presence of asymptomatic carriers can hamper control efforts and make it difficult to uncover the true details of the natural history of an infectious disease and to estimate the total infection prevalence. Here, we have shown that if carriage is not correctly incorporated into mathematical models used to inform control decisions, there is a risk that these models may produce substantially misleading predictions.

### 4.1. The impact of asymptomatic carriers on assessments of pathogen transmission and control

Our analysis revealed the possibility that certain interventions assessed as capable of interrupting transmission may actually lead to the opposite effect, or alternatively the effectiveness of an intervention may be underestimated, making it appear unviable in some circumstances. Specifically, poor estimates of a quantity which we interpret as the relative reproduction potential of carriers A can lead to incorrect assessments of interventions that alter the net rate at which infected hosts enter either the symptomatic or asymptomatic state. For example, a vaccine designed to reduce the incidence of symptomatically infected hosts by targeting pathogen strains associated with disease will always lead to a decrease in transmission and disease prevalence in the model if A<1. However, if A has been underestimated to a sufficient extent, then, as shown in figure 4*c*, this intervention may actually increase transmission and disease prevalence.

The same quantity also influences whether excluding asymptomatic carriers from our model either overestimates or underestimates the contribution of symptomatic infections to pathogen transmission. If A>1, asymptomatic carriers deplete the susceptible pool of hosts to a greater extent than symptomatic infectives. Failing to account for such asymptomatic carriers in mathematical models may lead to underestimates of transmission, and possibly also the prevalence of disease.

### 4.2. The impact of model structure on assessments of pathogen transmission and control

From our analysis it is clear that the threshold behaviour determined by the relative reproduction potential of carriers A is always present in our model when recovery from both carriage and symptomatic infection is allowed. If recovery is assumed to only occur from asymptomatic carriage, which is the assumption of some models of MRSA [14,17,20–22], then A<1 in the model, irrespective of the values of other parameters. Similarly, if recovery is only allowed from symptomatic infection, which is assumed in some models of abstract pathogens [28,29], then A>1 in the model irrespective of the values of other parameters.

Therefore, the threshold behaviour due to A is not a universal property of mathematical models with asymptomatic carriage. An implication of this is that control recommendations based on the wrong carriage model could be misleading. Specifically, if recommendations are based on a model that does not exhibit threshold behaviour and the model reflecting the true dynamics of the pathogen does, then there is the risk that interventions predicted to succeed will actually fail, or conversely that effective interventions will be negatively assessed. In our model this could happen if it is assumed that recovery is only allowed from carriage and not symptomatic infection. In this case, an intervention that reduces the rate of symptomatic infections will always be predicted to reduce transmission. However, if recovery is in fact possible from both infection states, then there is the possibility that this intervention will have the opposite effect and increase transmission (as shown in figure 4*b*).

Our results highlight the importance of accurately characterizing the relationship between asymptomatic carriers and symptomatic infections through a purposive process incorporating the clarification of the dominant modes of pathogen transmission, the conduct of carriage studies, seroepidemiology and active contact tracing to inform the correct attribution of infectious states.

### 4.3. Threshold behaviour in other mathematical models that incorporate asymptomatic carriers

The threshold behaviour that characterizes our model has been identified in other models of pathogen transmission incorporating asymptomatic carriage [28,30]. In these studies, it was found that decreasing the proportion of new cases of symptomatic infection will only decrease a pathogen’s basic reproduction number [28] and total prevalence [30] if carrier infectivity is sufficiently low. Here, we have shown that this threshold behaviour also applies to changes in the rate that established infections either become symptomatic or asymptomatic, and that it is the value of the relative reproduction potential of carriers, not just their relative infectivity, which is the critical determinant of this threshold behaviour.

Another threshold behaviour identified in models with asymptomatic carriers and with implications for control is a phenomenon known as *subcritical persistence*, which can allow an infectious disease to persist in a host population post intervention under certain conditions where, otherwise, it would have disappeared. In the models described in [30,31], subcritical persistence can occur if carriers have sufficiently high infectivity. In the model presented here, however, subcritical persistence is not possible [32].

### 4.4. The contribution of asymptomatic carriers to observations of pathogen transmission and control

A key result of our study is that interventions that alter the frequency of symptomatically infected hosts can lead to unintuitive outcomes in the presence of asymptomatic carriers. Vaccines targeted against a multi-strain pathogen almost invariably target only a proportion of strains. This partial coverage has the potential to change the balance of asymptomatic to symptomatic infection episodes, with implications for population-level vaccine impact.

The PCV7 *Streptococcus pneumoniae* vaccine, for example, targeted the seven most-prevalent strains associated with invasive disease when it was first added to routine childhood vaccination schedules in various populations. Numerous studies documented the subsequent declining rates of asymptomatic carriage of PCV7 serotypes and of overall invasive pneumococcal disease (IPD) among young children [23,33], although the overall incidence of asymptomatic carriage remained relatively stable [34]. Overall reductions in the incidence of IPD were also observed in non-vaccinated adults [23,33], although these were accompanied by increases in the overall rates of asymptomatic carriage [34]. The mechanisms behind the extended response to the PCV7 vaccine beyond the vaccinated populations which affected childhood and adult asymptomatic carriage and IPD rates differently are not well understood. However, it has been suggested that both serotype replacement and a decrease in herd immunity from reduced exposure to asymptomatic carriers may play a role in the population-level impact of the PCV7 vaccine [35,36]. Similar mechanisms are also suggested to be behind the extended response beyond the vaccinated populations to the subsequently introduced 13-valent *S. pneumoniae* vaccine [33]. Recently, IPD incidence has begun to increase in populations in the United Kingdom, particularly among persons 5–64 and greater than 65 years of age, and despite the ever-increasing proportion of the population obtaining direct protection through vaccination [37]. Serotype replacement, in conjunction with changes to the invasiveness of the non-vaccine strains, is a possible explanation [37].

In addition to the action of interventions, the frequency of symptomatic infections may also be altered by different host settings. Accordingly, patterns of pathogen transmission may also be expected to vary between host settings. MRSA and *Streptococcus pyogenes*, for example, have mostly commensal interactions with humans and occasionally cause symptomatic skin infections. When such pathogens are transmitted in environments with high rates of host injury (e.g. healthcare settings or regions where other contagious skin diseases like human scabies are endemic [38]), it is conceivable that infected hosts will probably have an increased chance of developing symptoms compared to other environments with lower host-injury rates and lower chances of hosts having breakages in their skin [15]. Our analytical results suggest that different patterns of pathogen transmission may be occurring in these different host settings, and that the effect of controls may also vary across host settings.

For example, asymptomatic carriers are thought to be more important for the transmission of healthcare-associated MRSA (HA-MRSA) than symptomatically infected hosts, reflected in the effectiveness of patient decolonization and improved hand hygiene compliance of healthcare workers for control [39]. Outside healthcare settings, community-associated MRSA (CA-MRSA) has emerged globally and independently of HA-MRSA [40]. One explanation for the sustained transmission of CA-MRSA outside healthcare settings is that it has a higher fitness in the community than HA-MRSA [40]. Our results indicate that the high reproduction potential of asymptomatic carriers is also likely to be playing a role in the transmission of CA-MRSA in the community. Thus carriers should be targets for control, as has been suggested previously [15].

*Streptococcus pyogenes* is characterized by a large difference in the prevalence of infections in high-income settings versus settings of poverty. In fact, children in remote Indigenous communities in northern Australia, New Zealand and the Pacific Islands have the highest prevalence of skin sores caused by *S. pyogenes* in the world [38]. These communities are characterized by higher host-injury rates and poorer host health compared to communities where *S. pyogenes* has a much lower prevalence. From our analytical results, we can deduce that asymptomatic carriers of *S. pyogenes* have a lower reproduction potential than symptomatically infected hosts, because increasing the proportion of symptomatic infections across host settings corresponds to an increase in prevalence.

### 4.5. Limitations and future work

Our proposed model of infectious disease transmission with asymptomatic carriers has a number of limitations. First, it applies to only those pathogens for which asymptomatic carriers are infectious, clearance of infection is possible and does not confer lifelong immunity, and infection does not alter the death rate of hosts. These restrictions mean that our results are not relevant to two of humanity’s leading causes of death attributable to a single infectious agent – *Mycobacterium tuberculosis* and HIV [41]. A natural extension of our work is to consider how relaxing these constraints affects our study’s conclusions to gain insight into the transmission of these important human pathogens. Second, our model assumes the homogeneous mixing of hosts and it does not consider strain diversity. However, for pathogens such as *S. pneumoniae* and *S. pyogenes* age-assortative mixing and strain diversity are likely to be key drivers of observed transmission dynamics [36,42]. Therefore, it is also of interest to explore the implications of asymptomatic carriage in more complex model structures.

## Supplementary Material

Supplementary appendices and figures

## Supplementary Material

Figure 3 Matlab code

## Supplementary Material

Figures 4 and S3 Matlab code

## Supplementary Material

Figure S2 Matlab code

## Supplementary Material

Figure S4 Matlab code

## Data accessibility

The code supporting this article has been uploaded as part of the electronic supplementary material.
