# Supplementary material for: Implications of asymptomatic carriers for infectious disease transmission and control
Source: R Soc Open Sci. 2018 Feb 14;5(2):172341. doi: 10.1098/rsos.172341 (PMC5830799; doi:10.1098/rsos.172341)
Supplement: Supplementary appendices and figures [file rsos172341supp1.pdf]

Electronic supplementary material

Implications of asymptomatic carriers for infectious disease transmission and control.

Rebecca H. Chisholm, Patricia T. Campbell, Yue Wu, Steven Y. C. Tong, Jodie McVernon, Nicholas Geard

A Literature review

The literature review focuses exclusively on articles that dynamically model the epidemiology of pathogens causing both asymptomatic and symptomatic infections. Therefore, PubMed searches were carried out using the search terms "(models, theoretical [MeSH Terms]) AND dynamic* AND (analysis OR mathematic*) AND transmission AND infection* AND (carriage OR carrier* OR asymptomatic* OR coloni*)" identifying 132 articles. Of these, 101 articles were excluded because they were either reviews that did not present novel work, or because they presented either exclusively within-host models, models not incorporating symptomatic hosts, models including pathogen vectors, models where asymptomatic hosts are non-infectious, or statistical models. We then included a further eleven articles identified through manually searching. This left a total of 42 articles presenting 45 models of the transmission of various infectious diseases in the presence of asymptomatic carriers, which are summarised in Figure S1.

B Basic reproduction number

To determine the basic reproduction number we follow the next-generation method outlined in [1]. We construct the linearised infection subsystem from the system of equations (1) which describes only the production of new infecteds and changes in the states of already existing infecteds from the disease-free steady state (where $S = N$) so that

$$\begin{aligned} \frac{dC}{dt} &= (1-\alpha)\beta(I+\eta C) - (\xi\gamma+\tau)C + \omega I, \\ \frac{dI}{dt} &= \alpha\beta(I+\eta C) + \tau C - (\gamma+\omega)I. \end{aligned} \tag{S1}$$

We re-write the linearised infection subsystem in the form

$$\begin{pmatrix} \dot{C} \\ \dot{I} \end{pmatrix} = (\mathbf{T}+\mathbf{\Sigma})\begin{pmatrix} C \\ I \end{pmatrix}$$

where the matrix $\mathbf{T}$ incorporates all epidemiological events that lead to new infections, and all other events are included in the matrix $\mathbf{\Sigma}$ so that

$$\mathbf{T} = \begin{pmatrix} (1-\alpha)\beta\eta & (1-\alpha)\beta \\ \alpha\beta\eta & \alpha\beta \end{pmatrix}, \tag{S2}$$

and

$$\mathbf{\Sigma} = \begin{pmatrix} -\xi\gamma-\tau & \omega \\ \tau & -\gamma-\omega \end{pmatrix}. \tag{S3}$$

The next generation matrix $\mathbf{K}$ is then given by

$$\begin{aligned} \mathbf{K} &= -\mathbf{T}\mathbf{\Sigma}^{-1} \\ &= -\begin{pmatrix} (1-\alpha)\beta\eta & (1-\alpha)\beta \\ \alpha\beta\eta & \alpha\beta \end{pmatrix} \times \begin{pmatrix} -\frac{\gamma+\omega}{\gamma\tau+\xi\gamma(\gamma+\omega)} & -\frac{\omega}{\gamma\tau+\xi\gamma(\gamma+\omega)} \\ -\frac{\tau}{\gamma\tau+\xi\gamma(\gamma+\omega)} & -\frac{\xi\gamma+\tau}{\gamma\tau+\xi\gamma(\gamma+\omega)} \end{pmatrix} \\ &= \begin{pmatrix} \frac{(1-\alpha)\beta(\tau+\eta(\gamma+\omega))}{\gamma\tau+\xi\gamma(\gamma+\omega)} & -\frac{(\alpha-1)\beta(\xi\gamma+\tau+\eta\omega)}{\gamma\tau+\xi\gamma(\gamma+\omega)} \\ \frac{\alpha\beta(\tau+\eta(\gamma+\omega))}{\gamma\tau+\xi\gamma(\gamma+\omega)} & \frac{\alpha\beta(\xi\gamma+\tau+\eta\omega)}{\gamma\tau+\xi\gamma(\gamma+\omega)} \end{pmatrix}. \end{aligned}$$

The elements K_{ij} of the next generation matrix $\mathbf{K}$ can be interpreted as the expected number of new cases that an infected individual of type j causes among the susceptible individuals of type i, in a fully susceptible population. Thus, the expected number of new cases of infection due to a carrier is $K_{11} + K_{21}$. Similarly, a newly symptomatic host is expected to cause $K_{12} + K_{22}$ new cases in a fully susceptible population.

The dominant eigenvalue of the next generation matrix $\mathbf{K}$ is equal to the basic reproduction number $\mathcal{R}_0$. Hence,

$$\mathcal{R}_0 = \frac{\beta}{\gamma}\left(\frac{\alpha\xi\gamma + \tau + \eta[\gamma(1-\alpha)+\omega]}{\tau + \xi(\gamma+\omega)}\right). \tag{S4}$$

This expression for $\mathcal{R}_0$ can be decomposed into the form shown in equation (2) to reveal the contribution to $\mathcal{R}_0$ from symptomatic infections versus asymptomatic carriers.

In the main text we derive the following expression for the relative endemic prevalence $\mathcal{P}$ of asymptomatic carriers to symptomatic infections

$$\mathcal{P} = \frac{\omega + \gamma(1-\alpha)}{\tau + \xi\gamma\alpha}.$$

Here, we show how the basic reproduction number $\mathcal{R}_0$ depends on $\mathcal{P}$. From equation (S4) we see that

$$\begin{aligned}
\mathcal{R}_0 &= \frac{\beta}{\gamma}\left(\frac{\alpha\xi\gamma + \tau + \eta[\gamma(1-\alpha)+\omega]}{\tau + \xi(\gamma+\omega)}\right) \times \left(\frac{1/[\alpha\xi\gamma+\tau]}{1/[\alpha\xi\gamma+\tau]}\right), \\
&= \frac{\beta}{\gamma}\left(\frac{1 + \eta[\gamma(1-\alpha)+\omega]/[\alpha\xi\gamma+\tau]}{[\tau+\xi(\gamma+\omega)]/[\alpha\xi\gamma+\tau]}\right) = \frac{\beta}{\gamma}\left(\frac{1+\eta\mathcal{P}}{[\tau + \alpha\xi\gamma + \xi(\gamma+\omega) - \alpha\xi\gamma]/[\alpha\xi\gamma+\tau]}\right), \\
&= \frac{\beta}{\gamma}\left(\frac{1+\eta\mathcal{P}}{1+\xi[\gamma(1-\alpha)+\omega)]/[\alpha\xi\gamma+\tau]}\right) = \frac{\beta}{\gamma}\left(\frac{1+\eta\mathcal{P}}{1+\xi\mathcal{P}}\right) = \frac{\beta}{\gamma}\left(\frac{1+\xi\mathcal{P}\mathcal{A}}{1+\xi\mathcal{P}}\right).
\end{aligned}$$

Both $\mathcal{R}_0$ and $\mathcal{P}$ determine the respective endemic infection-type prevalences $\hat{P}_C$ and $\hat{P}_I$, which are found by setting the system of equations (1) to zero so that

$$0 = (1-\alpha)\lambda(\hat{C},\hat{I})\hat{S} - (\xi\gamma+\tau)\hat{C} + \omega\hat{I} \quad \text{and} \quad 0 = \alpha\lambda(\hat{C},\hat{I})\hat{S} + \tau\hat{C} - (\gamma+\omega)\hat{I}. \tag{S5}$$

By substituting $\hat{S} = N - \hat{I} - \hat{C}$, $\lambda(\hat{C},\hat{I}) = \beta(\eta\hat{C} + \hat{I})$ and $\hat{C} = \mathcal{P}\hat{I}$ into the second expression in equation (S5) we see that

$$0 = \alpha\beta(\eta\hat{I}\mathcal{P} + \hat{I})(N - \hat{I} - \hat{I}\mathcal{P})/N + \tau\mathcal{P}\hat{I} - (\gamma+\omega)\hat{I}.$$

Dividing by $\hat{I}$, this becomes

$$0 = \alpha\beta(\eta\mathcal{P}+1)[1 - (1+\mathcal{P})\hat{I}/N] + \tau\mathcal{P} - (\gamma+\omega),$$

which after some rearrangement gives

$$\begin{aligned}
\hat{P}_I = \frac{\hat{I}}{N} &= \left(\frac{1}{1+\mathcal{P}}\right)\left(\frac{\tau\mathcal{P} - (\gamma+\omega)}{\alpha\beta(\eta\mathcal{P}+1)} + 1\right) = \left(\frac{1}{1+\mathcal{P}}\right)\left(1 - \frac{1}{\mathcal{R}_0}\left(\frac{\gamma+\omega-\tau\mathcal{P}}{\alpha\gamma(1+\xi\mathcal{P})}\right)\right), \\
&= \left(\frac{1}{1+\mathcal{P}}\right)\left(1 - \frac{1}{\mathcal{R}_0}\left(\frac{\gamma+\omega-\tau\mathcal{P}}{\alpha\gamma(1+\xi\mathcal{P})}\right)\frac{(\tau+\xi\gamma\alpha)}{(\tau+\xi\gamma\alpha)}\right) \\
&= \left(\frac{1}{1+\mathcal{P}}\right)\left(1 - \frac{1}{\mathcal{R}_0}\left(\frac{(\gamma+\omega)(\tau+\xi\gamma\alpha) - \tau(\omega+\gamma(1-\alpha))}{\alpha\gamma(\tau+\xi\gamma\alpha+\xi(\omega+\gamma(1-\alpha)))}\right)\right) \\
&= \left(\frac{1}{1+\mathcal{P}}\right)\left(1 - \frac{1}{\mathcal{R}_0}\left(\frac{(\gamma+\omega)\xi\gamma\alpha + \tau\gamma\alpha}{\alpha\gamma(\tau+\xi(\omega+\gamma))}\right)\right) = \left(\frac{1}{1+\mathcal{P}}\right)\left(1 - \frac{1}{\mathcal{R}_0}\right),
\end{aligned}$$

and so

$$\hat{P}_C = \mathcal{P}\hat{P}_I = \left(\frac{\mathcal{P}}{1+\mathcal{P}}\right)\left(1 - \frac{1}{\mathcal{R}_0}\right).$$

One could also substitute $\hat{S} = N - \hat{I} - \hat{C}$, $\lambda(\hat{C},\hat{I}) = \beta(\eta\hat{C} + \hat{I})$ and $\hat{C} = \mathcal{P}\hat{I}$ into the first expression in equation (S5) to obtain the same result.

Finally, we show that $\mathcal{R}_0$ is a threshold for global stability of the disease-free steady state to rule out the possibility of backward bifurcation. Following the method outlined in [2], we re-write the system of equations (1) in the form

$$x' = \mathcal{F}(x,y) - \mathcal{V}(x,y), \quad y' = g(x,y). \tag{S6}$$

Here, $'$ denotes differentiation with respect to time; $x = (C, I)^T \in \mathbb{R}^2$ and $y = S \in \mathbb{R}$; $\mathcal{F} = (\mathcal{F}_1, \mathcal{F}_2)^T$ and $\mathcal{V} = (\mathcal{V}_1, \mathcal{V}_2)^T$, where $\mathcal{F}_i$ represents the rate of new infections in compartment i; and $\mathcal{V}_i$ represents the transition terms in compartment i such that

$$\mathcal{F} = \begin{pmatrix} (1-\alpha)\beta S(I+\eta C)/N \\ \alpha\beta S(I+\eta C)/N \end{pmatrix}, \quad \text{and} \quad \mathcal{V} = \begin{pmatrix} \gamma\xi C + \tau C - \omega I \\ \gamma I - \tau C + \omega I \end{pmatrix}. \tag{S7}$$

We note that the feasible region $\Gamma = \{(S, I, C) \in \mathbb{R}^3_+ | S + I + C = N\}$ is positively invariant with respect to (S6). The model (S6) always admits a disease-free equilibrium $(S, I, C) = (N, 0, 0) \in \Gamma$. An endemic equilibrium $(S, I, C) = (\hat{S}, \hat{I}, \hat{C}) \in \Gamma$ exists if and only if the basic reproduction number $\mathcal{R}_0 > 1$ with $\hat{S} = N/\mathcal{R}_0$, $\hat{I} = N(1 - 1/\mathcal{R}_0)/(1 + \mathcal{P})$ and $\hat{C} = N\mathcal{P}(1 - 1/\mathcal{R}_0)/(1 + \mathcal{P})$ where $\mathcal{P} = (\omega + \gamma(1-\alpha))/(\tau + \xi\gamma\alpha)$.

We set

$$f(x, y) = (\mathbf{T} + \mathbf{\Sigma})x - \mathcal{F}(x, y) + \mathcal{V}(x, y) \tag{S8}$$

$$= \begin{pmatrix} (1-\alpha)\beta(I+\eta C)(1 - S/N) \\ \alpha\beta(I+\eta C)(1 - S/N) \end{pmatrix}, \tag{S9}$$

where $\mathbf{T}$ and $\mathbf{\Sigma}$ are defined as above, which satisfies $f(x, y) \geq 0$ with $f(x, N) = 0$ in Γ. Since $\mathbf{T} \geq 0$, $-\mathbf{\Sigma}^{-1} \geq 0$, and

$$-\mathbf{\Sigma}^{-1}\mathbf{T} = \begin{pmatrix} \frac{\gamma+\omega}{\gamma\tau+\xi\gamma(\gamma+\omega)} & \frac{\omega}{\gamma\tau+\xi\gamma(\gamma+\omega)} \\ \frac{\tau}{\gamma\tau+\xi\gamma(\gamma+\omega)} & \frac{\xi\gamma+\tau}{\gamma\tau+\xi\gamma(\gamma+\omega)} \end{pmatrix} \times \begin{pmatrix} (1-\alpha)\beta\eta & (1-\alpha)\beta \\ \alpha\beta\eta & \alpha\beta \end{pmatrix}$$

$$= \begin{pmatrix} \frac{\beta(\omega+\gamma(1-\alpha))}{\gamma\tau+\xi\gamma(\gamma+\omega)} & \frac{\beta\eta(\omega+\gamma(1-\alpha))}{\gamma\tau+\xi\gamma(\gamma+\omega)} \\ \frac{\beta(\tau+\alpha\gamma\xi)}{\gamma\tau+\xi\gamma(\gamma+\omega)} & \frac{\beta\eta(\tau+\alpha\gamma\xi)}{\gamma\tau+\xi\gamma(\gamma+\omega)} \end{pmatrix}$$

is irreducible, then by Theorem 2.2 in [2], the disease-free equilibrium $(S, I, C) = (N, 0, 0)$ is globally asymptotically stable in Γ provided that $\mathcal{R}_0 < 1$.

C Model accounting for host demography

Accounting for demography in the system of equations that describe our model is straightforward. We simply replace the recovery rates γ and $\xi\gamma$ with the respective rates $\gamma + \mu$ and $\xi\gamma + \mu$ that include a contribution μ which is the per capita rate of host death in the model for each host type. Here we are assuming that this death rate balances the birth rate so that the total population size N remains constant.

In this case, the basic reproduction number becomes,

$$\mathcal{R}_0 = \beta \left(\frac{\alpha(\xi\gamma + \mu) + \tau + \eta[(\gamma + \mu)(1-\alpha) + \omega]}{\tau(\gamma + \mu) + (\xi\gamma + \mu)(\gamma + \mu + \omega)} \right), \tag{S10}$$

the relative reproduction potential of asymptomatic carriers is

$$\mathcal{A} = \frac{\eta(\gamma + \mu)}{\xi\gamma + \mu}, \tag{S11}$$

and the relative endemic prevalence of asymptomatic carriers is

$$\mathcal{P} = \frac{\omega + (1-\alpha)(\gamma + \mu)}{\tau + \alpha(\xi\gamma + \mu)}. \tag{S12}$$

The form of all other equations in the main text remain the same with the above expressions for $\mathcal{R}_0$, $\mathcal{A}$, and $\mathcal{P}$ substituted in. Hence, our results regarding the impacts of carriage on predictions from epidemiological models do not change when we consider pathogens whose timescale of disease is comparable to the timescale of host demographic change.

D Additional calculations

The effect of asymptomatic carriers on predictions of the endemic prevalence of symptomatic infections $\hat{P}_I$. Here we compare the endemic prevalence of symptomatic infections predicted using the full model $\hat{P}_I$ to that of the SIS model $\hat{P}_{\text{SIS}}$ when $\beta_{\text{SIS}} = \beta$ and $\gamma_{\text{SIS}} = \gamma$. We only consider the case where

$\mathcal{R}_{0,\mathrm{SIS}} > 1$ and $\mathcal{R}_0 > 1$ so that the endemic equilibrium in both models is stable and biologically meaningful. It is straightforward to show that $\hat{P}_{\mathrm{SIS}} > \hat{P}_I$ when

$$(1 + \mathcal{A}\mathcal{P}\xi)(\mathcal{R}_{0,\mathrm{SIS}} - 1) + (1 - \mathcal{A})\xi > 0. \tag{S13}$$

This condition always holds true when $\mathcal{A} < 1$. Therefore, when the host-specific reproduction number of asymptomatic carriers is less than that of symptomatically infected hosts, excluding asymptomatic carriers from our model will always lead to overestimating the endemic prevalence of symptomatic infections. If instead $\mathcal{A} > 1$, then it is possible that the SIS model will underestimate the endemic level of disease.

How can infrequent asymptomatic carriers play a key role in pathogen transmission? To understand how asymptomatic carriers can contribute the most to transmission at endemic equilibrium while remaining less prevalent than symptomatically infected hosts we consider the endemic incidence of infection $\hat{\Omega}$, which is the total number of new cases of infection (both symptomatic infection and asymptomatic carriage) per unit time at endemic equilibrium. In our model, it is identified from the system of equations (1) as

$$\hat{\Omega} = \hat{\Omega}_C + \hat{\Omega}_I \tag{S14}$$

where $\hat{\Omega}_C$ is the contribution to the endemic incidence of infection by asymptomatic carriers, and $\hat{\Omega}_I$ is the contribution by symptomatic infectives such that

$$\hat{\Omega}_C = \beta\eta\hat{S}\hat{C}/N \quad \text{and} \quad \hat{\Omega}_I = \beta\hat{S}\hat{I}/N.$$

Clearly, asymptomatic carriers contribute more to the endemic incidence when $\hat{\Omega}_C > \hat{\Omega}_I$ which holds true when $\eta\mathcal{P} > 1$. Thus, asymptomatic carriers can be responsible for the majority of endemic transmissions whilst remaining in the minority (in terms of prevalence) when

$$\eta\mathcal{P} > 1 > \mathcal{P}, \tag{S15}$$

which is only possible if asymptomatic carriers have a sufficiently greater infectivity than symptomatic infectives. Hence, even if the prevalence of asymptomatic carriers is grossly outweighed by that of symptomatic infectives, it is still possible for asymptomatic carriers to be playing a key role in pathogen transmission.

The impact of control interventions on the endemic prevalence of symptomatic infections $\hat{P}_I$. Here we explore how interventions may change the endemic prevalence of symptomatic infections $\hat{P}_I$. Again, we only consider the case where $\mathcal{R}_0 > 1$ so that the endemic equilibrium is stable and biologically meaningful. It is straightforward to show that

$$\frac{\partial \hat{P}_I}{\partial x} = -\left(\frac{1}{1+\mathcal{P}}\right)^2 \frac{\partial \mathcal{P}}{\partial x}\left(1 - \frac{1}{\mathcal{R}_0}\right) + \left(\frac{1}{1+\mathcal{P}}\right)\left(\frac{1}{\mathcal{R}_0}\right)^2 \frac{\partial \mathcal{R}_0}{\partial x}. \tag{S16}$$

Therefore $\partial \hat{P}_I/\partial x > 0$ when

$$\left(\frac{1}{1+\mathcal{P}}\right)\frac{\partial \mathcal{P}}{\partial x} < \left(\frac{1}{\mathcal{R}_0^2 - \mathcal{R}_0}\right)\frac{\partial \mathcal{R}_0}{\partial x}. \tag{S17}$$

For the parameters $x \in \{\beta, \eta\}$, condition (S17) always holds true since $\partial\mathcal{P}/\partial x = 0$ and $\partial\mathcal{R}_0/\partial x > 0$ for all $x \in \{\beta, \eta\}$. This makes intuitive sense since increasing the infectivity of either infection type (through increasing β or η) will always result in more cases of both infection types. For all other parameters, the effect of interventions on the endemic prevalence of symptomatic infections $\hat{P}_I$ is more complicated.

For the parameters $x \in \{\gamma, \xi\gamma\}$, both $\partial\mathcal{R}_0/\partial x < 0$ and $\partial\mathcal{P}/\partial x < 0$ always holds true. Therefore, an intervention that alters the clearance rate of carriage or symptomatic infection (that is, either γ or $\xi\gamma$) may either increase or decrease $\hat{P}_I$, depending on whether condition (S17) is satisfied.

For $x \in \{\omega\}$, $\partial\hat{P}_I/\partial x < 0$ when $\mathcal{A} < 1$ since $\partial\mathcal{P}/\partial x > 0$ always holds true and $\partial\mathcal{R}_0/\partial x < 0$ for $x \in \{\omega\}$ if and only if $\mathcal{A} < 1$. However, if $\mathcal{A} > 1$ then an intervention that alters ω may either increase or decrease $\hat{P}_I$, depending on whether condition (S17) is satisfied. For $x \in \{\tau, \alpha\}$, $\partial\hat{P}_I/\partial x > 0$ when $\mathcal{A} < 1$ since $\partial\mathcal{P}/\partial x < 0$ always holds true and $\partial\mathcal{R}_0/\partial x > 0$ for $x \in \{\tau, \alpha\}$ if and only if $\mathcal{A} < 1$. However, if $\mathcal{A} > 1$ then an intervention that alters τ or α may either increase or decrease $\hat{P}_I$, depending on whether condition (S17) is satisfied. Therefore, when asymptomatic carriers have less potential for infection reproduction than symptomatic infectives (so that $\mathcal{A} < 1$), decreasing the relative frequency of appearance of symptomatic infectives (by decreasing τ or α, or increasing ω) will always result in a lower endemic prevalence of disease $\hat{P}_I$. If, on the other hand, asymptomatic carriers have a higher reproduction potential (so that $\mathcal{A} > 1$), then decreasing the relative frequency of appearance of symptomatic infectives may actually increase the endemic prevalence of disease $\hat{P}_I$.

E Parameter values used for plots in figures

Figure 3: $N = 1000$, $\gamma = 0.1$, $\beta = 0.13$, $\alpha = 0.3$, $\tau = 0.05$, and (a) $\xi = 0.1$, $\omega = 0.2$, $\mathcal{A} = 0.8$, $\eta\mathcal{P} = 0.41$; (b) $\xi = 0.1$, $\omega = 0.2$, $\mathcal{A} = 1.5$, $\eta\mathcal{P} = 0.76$; and (c) $\xi = 4$, $\omega = 0.01$, $\mathcal{A} = 1.5$, $\eta\mathcal{P} = 2.82$.

Figure 4: $N = 1000$, $C(0) = 10$, $I(0) = 0$, $\gamma = 0.1$, (a) $\mathcal{A} \in \{0.75, 1.2\}$, $\omega \in \{0.1, 0.3\}$, $\tau = 0.1$, $\alpha = 0.1$, $\xi = 0.6$, $\beta = 0.12$; (b) $\mathcal{A} \in \{0.7, 1.2\}$, $\tau \in \{0.06, 0.02\}$, $\omega = 0.1$, $\alpha = 0.1$, $\xi = 0.6$, $\beta = 0.13$; (c) $\mathcal{A} \in \{0.7, 1.4\}$, $\alpha \in \{0.7, 0.2\}$, $\tau = 0.06$, $\omega = 0.1$, $\xi = 0.5$, $\beta = 0.12$.

Figure S2: $\beta = 0.25$, $\xi = 0.05$, $\gamma = 0.2$, $\mathcal{A} \in [0, 2]$, $\alpha = 0.4$, $\omega = 0.2$, $\tau \in [0, 0.1]$.

Figure S3: $N = 1000$, $C(0) = 10$, $I(0) = 0$, $\gamma = 0.1$, (a–c) $\mathcal{A} \in \{0.75, 1.2\}$, $\omega \in \{0.1, 0.3\}$, $\tau = 0.1$, $\alpha = 0.1$, $\xi = 0.6$, $\beta = 0.12$; (d–f) $\mathcal{A} \in \{0.7, 1.2\}$, $\tau \in \{0.06, 0.02\}$, $\omega = 0.1$, $\alpha = 0.1$, $\xi = 0.6$, $\beta = 0.13$; (g–i) $\mathcal{A} \in \{0.7, 1.4\}$, $\alpha \in \{0.7, 0.2\}$, $\tau = 0.06$, $\omega = 0.1$, $\xi = 0.5$, $\beta = 0.12$.
Figure S4: $N = 1000$, $C(0) = 10$, $I(0) = 0$, $\gamma = 0.015$, $\mathcal{A} = 80$, $\omega = 0.1$, $\tau = 0.7$, $\alpha \in \{0.6, 0.5\}$, $\xi = 0.05$, $\beta = 0.01$.

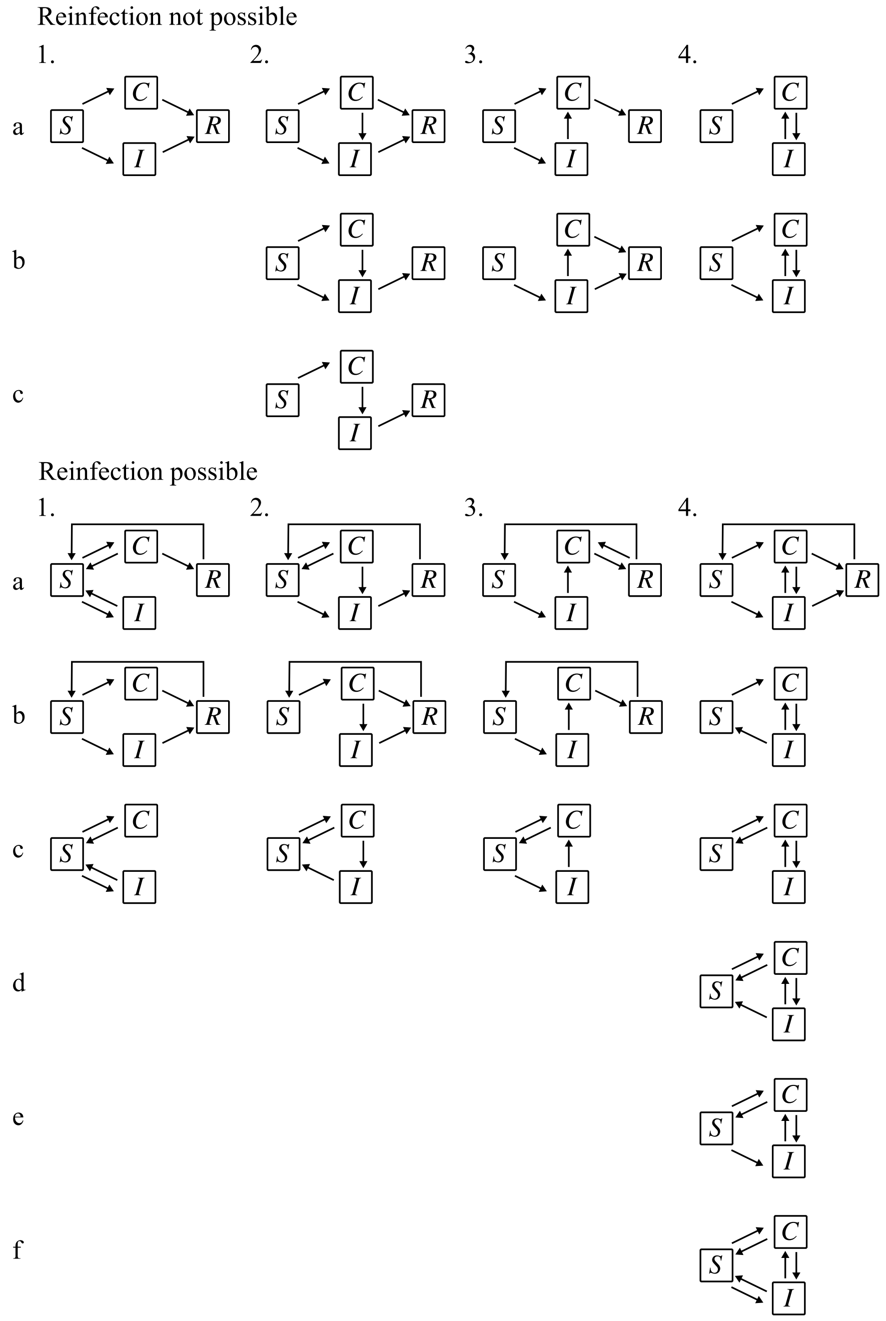

Figure S1: Initial categorisation of the generic carriage models identified in the literature review. Models are separated into those which do not allow reinfection (top) and those that do allow reinfection (bottom). Models are then sorted according to the transitions between C and I so that models in column 1 have no transitions between C and I, column 2 has models with the $C \to I$ transition only, column 3 has models with the $I \to C$ transition only, and column 4 has models with both $C \to I$ and $I \to C$ transitions. The articles identified in the review using the model structures shown here are as follows. *Reinfection not possible:* (1a) [3, 4, 5, 6, 7] (2a) [3] (2b) [8, 9] (2c) [3] (3a) [10, 11] (3b) [12, 13, 14, 15] (4a) [16] (4b) [17]. *Reinfection possible:* (1a) [18] (1b) [19, 20, 21, 22, 23] (1c) [24, 25, 26, 27, 28, 29, 30] (2a) [31] (2b) [21] (2c) [32] (3a) [33] (3b) [34] (3c) [35] (4a) [36] (4b) [37] (4c) [38, 39, 40] (4d) [41] (4e) [42, 43] (4f) [44].

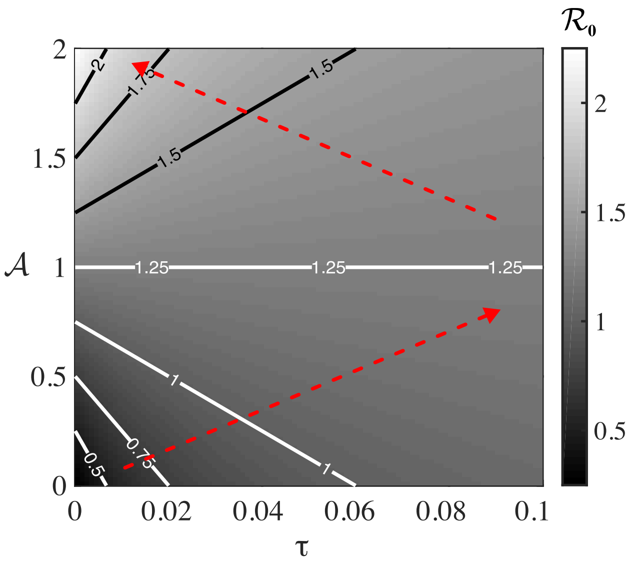

Figure S2: The basic reproduction number $\mathcal{R}_0$ is shown as a function of the relative reproduction potential of asymptomatic carriers $\mathcal{A}$ (vertical axis) and the progression rate to symptomatic infection τ (horizontal axis). The dashed red lines indicate directions where $\mathcal{R}_0$ is increasing, while the solid white and black lines indicate contours where $\mathcal{R}_0$ is constant. It is clear that the slope of $\mathcal{R}_0$ in the direction of τ is dependent on whether $\mathcal{A} > 1$ or $\mathcal{A} < 1$. Parameter values are provided in appendix E.

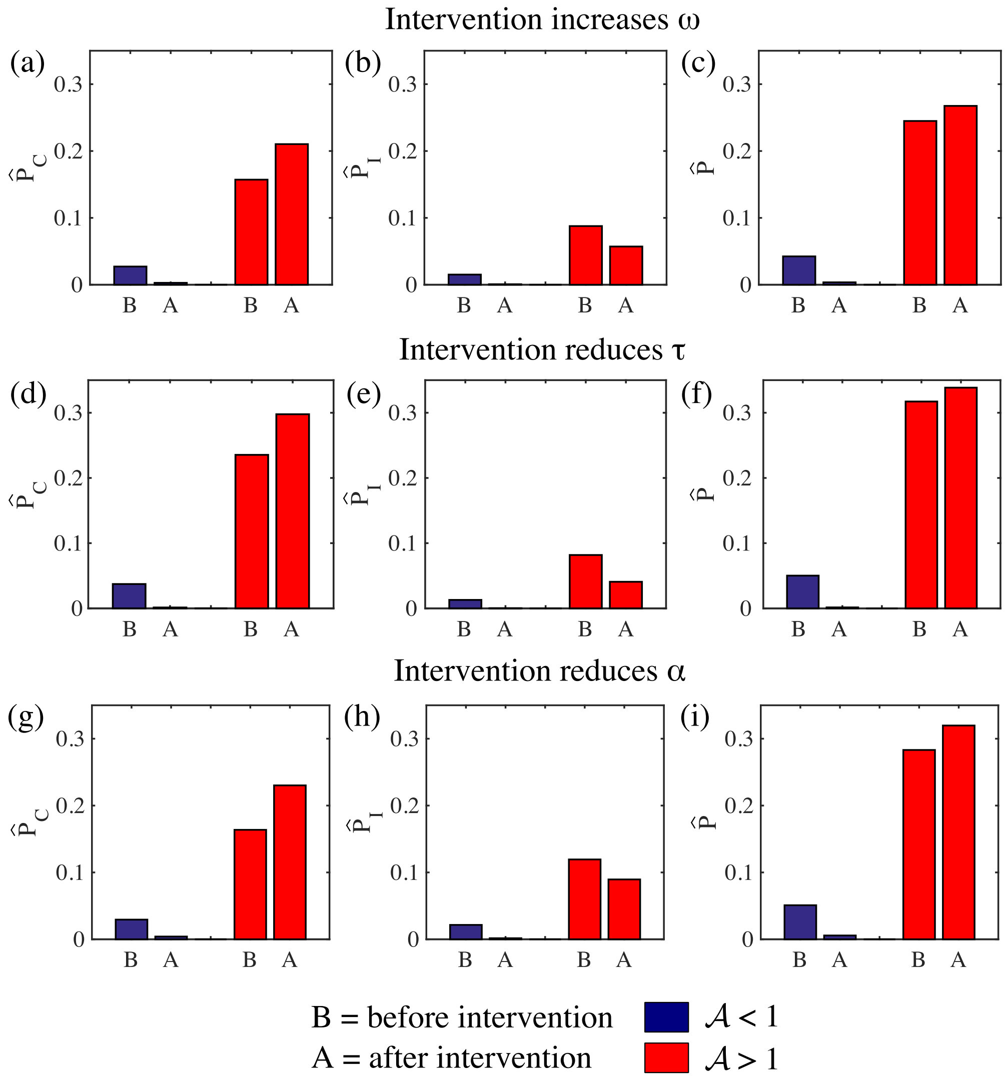

Figure S3: The endemic prevalence of asymptomatic carriers $\hat{P}_C$ (a,d,g), symptomatic infections $\hat{P}_I$ (b,e,h), and total endemic prevalence $\hat{P}$ (c,f,i) is shown before (B) and after (A) an intervention when $\mathcal{A} < 1$ (blue) and when $\mathcal{A} > 1$ (red). These scenarios correspond to those shown in Figure 4 in the main text. (a–c) The intervention increases ω; (d–f) The intervention decreases τ; and (g–i) The intervention decreases α. Parameter values are provided in appendix E.

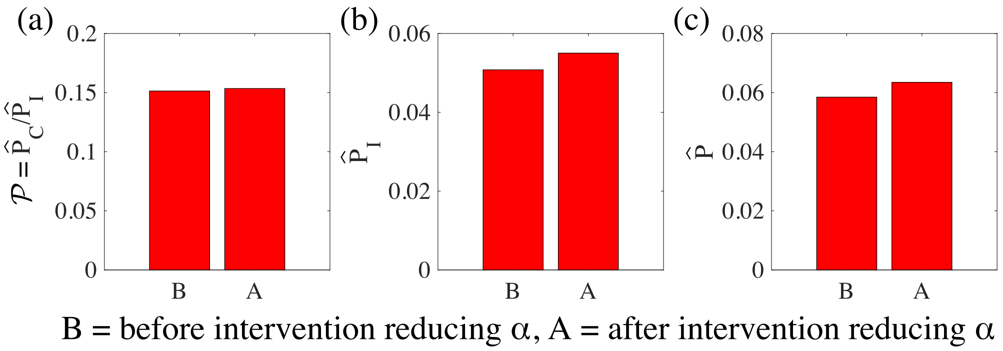

Figure S4: The endemic relative prevalence of asymptomatic carriers compared to symptomatic infections $\mathcal{P}$ (a), endemic prevalence of symptomatic infections $\hat{P}_I$ (b), and total endemic prevalence $\hat{P}$ (c) are shown before (B) and after (A) an intervention that reduces α. Here, the intervention increases the relative prevalence of asymptomatic carriers and the absolute prevalence of symptomatic infections. Parameter values are provided in appendix E.

References

[1] O. Diekmann, J. A. P. Heesterbeek, M. G. Roberts, The construction of next-generation matrices for compartmental epidemic models, J R Soc Interface 7 (2010) 873–885.

[2] Z. Shuai, P. Van Den Driessche. Global stability of infectious disease models using Lyapunov functions, J Appl Math 73 (4) (2013) 1513–1532.

[3] K. Ejima, K. Aihara, H. Nishiura, The impact of model building on the transmission dynamics under vaccination: observable (symptom-based) versus unobservable (contagiousness-dependent) approaches, PLoS One 8 (4) (2013) e62062.

[4] S.-B. Hsu, Y.-H. Hsieh, On the role of asymptomatic infection in transmission dynamics of infectious diseases, Bull Math Biol 70 (1) (2008) 134–55.

[5] J. T. Kemper, Identification of silent infections in SIR epidemics, Bull Math Biol 43 (3) (1981) 249–257.

[6] T. Van Effelterre, G. Dos Santos, V. Shinde, Twin peaks: A/H1N1 pandemic influenza virus infection and vaccination in Norway, 2009-2010, PLoS One 11 (3) (2016) e0151575.

[7] Z.-Q. Xia, J. Zhang, Y.-K. Xue, G.-Q. Sun, Z. Jin, Modeling the transmission of Middle East Respiratory Syndrome corona virus in the Republic of Korea, PLoS One 10 (12) (2015) e0144778.

[8] D. Kalajdzievska, M. Y. Li, Modeling the effects of carriers on transmission dynamics of infectious diseases, Math Biosci Eng 8 (3) (2011) 711–722.

[9] M. L. M. Manyombe, J. Mbang, J. Lubuma, B. Tsanou, Global dynamics of a vaccination model for infectious diseases with asymptomatic carriers, Math Biosci Eng 13 (4) (2016) 813.

[10] P. Ezanno, M. Lesnoff, A metapopulation model for the spread and persistence of contagious bovine pleuropneumonia (CBPP) in African sedentary mixed crop-livestock systems, J Theor Biol 256 (4) (2009) 493–503.

[11] M. Lesnoff, G. Laval, P. Bonnet, K. Chalvet-Monfray, R. Lancelot, F. Thiaucourt, A mathematical model of the effects of chronic carriers on the within-herd spread of contagious bovine pleuropneumonia in an African mixed crop-livestock system, Prev Vet Med 62 (2) (2004) 101–17.

[12] W. J. Edmunds, G. F. Medley, D. J. Nokes, The transmission dynamics and control of hepatitis B virus in The Gambia, Stat Med 15 (20) (1996) 2215–33.

[13] M. Kretzschmar, G. A. de Wit, L. J. M. Smits, M. J. W. van de Laar, Vaccination against hepatitis B in low endemic countries, Epidemiol Infect 128 (2) (2002) 229–44.

[14] L. Zou, W. Zhang, S. Ruan, Modeling the transmission dynamics and control of hepatitis B virus in China, J Theor Biol 262 (2) (2010) 330–8.

[15] M. G. Roberts, The pluses and minuses of R_0, J R Soc Interface 4 (2007) 949–961.

[16] C. P. Bhunu, W. Garira, G. Magombedze, Mathematical analysis of a two strain HIV/AIDS model with antiretroviral treatment, Acta Biotheor 57 (3) (2009) 361–81.

[17] B. Y. Lee, R. R. Bailey, K. J. Smith, R. R. Muder, E. S. Strotmeyer, G. J. Lewis, P. J. Ufberg, Y. Song, L. H. Harrison, Universal methicillin-resistant *Staphylococcus aureus* (MRSA) surveillance for adults at hospital admission: an economic model and analysis, Infect Control Hosp Epidemiol 31 (6) (2010) 598–606.

[18] C. L. Althaus, J. C. M. Heijne, A. Roellin, N. Low, Transmission dynamics of *Chlamydia trachomatis* affect the impact of screening programmes, Epidemics 2 (3) (2010) 123–31.

[19] B. M. Althouse, S. V. Scarpino, Asymptomatic transmission and the resurgence of *Bordetella pertussis*, BMC Med 13 (1) (2015) 146.

[20] Y. Ma, M. Liu, Q. Hou, J. Zhao, Modelling seasonal HFMD with the recessive infection in Shandong, China, Math Biosci Eng 10 (4) (2013) 1159–71.

[21] M. Robinson, N. I. Stilianakis, A model for the emergence of drug resistance in the presence of asymptomatic infections, Math Biosci 243 (2) (2013) 163–177.

[22] D. M. Vickers, A. M. Anonychuk, P. De Wals, N. Demarteau, C. T. Bauch, Evaluation of serogroup C and ACWY meningococcal vaccine programs: projected impact on disease burden according to a stochastic two-strain dynamic model, Vaccine 33 (1) (2015) 268–75.

[23] J. Wang, Y. Xiao, R. A. Cheke, Modelling the effects of contaminated environments on HFMD infections in mainland China, Biosystems 140 (2016) 1–7.

[24] F. J. Bowden, G. P. Garnett, *Trichomonas vaginalis* epidemiology: parameterising and analysing a model of treatment interventions, Sex Transm Infect 76 (4) (2000) 248–56.

[25] K. L. Cooke, Models for endemic infections with asymptomatic cases. I. One group, Math Mod 3 (1) (1982) 1–15.

[26] G. P. Garnett, K. J. Mertz, L. Finelli, W. C. Levine, M. E. S. Louis, The transmission dynamics of gonorrhoea: modelling the reported behaviour of infected patients from Newark, New Jersey, Phil.Trans R Soc Lond B 354 (1999) 787–797.

[27] A. Hazel, S. Marino, C. Simon, An anthropologically based model of the impact of asymptomatic cases on the spread of *Neisseria gonorrhoeae*, J R Soc Interface 12 (106) (2015) 20150067.

[28] J. T. Kemper, The effects of asymptomatic attacks on the spread of infectious disease: a deterministic model, Bull Math Biol 40 (6) (1978) 707–718.

[29] B. R. Morin, L. Medina-Rios, E. T. Camacho, C. Castillo-Chavez, Static behavioral effects on gonorrhea transmission dynamics in a MSM population, J Theor Biol 267 (1) (2010) 35–40.

[30] S. J. Snedecor, D. R. Strutton, V. Ciuryla, E. J. Schwartz, M. F. Botteman, Transmission-dynamic model to capture the indirect effects of infant vaccination with Prevnar (7-valent pneumococcal conjugate vaccine (PCV7)) in older populations, Vaccine 27 (34) (2009) 4694–703.

[31] C. Hogea, T. van Effelterre, C. J. Acosta, A basic dynamic transmission model of *Staphylococcus aureus* in the US population, Epidemiol Infect 142 (3) (2014) 468–78.

[32] S. Tartof, A. Cohn, F. Tarbangdo, M. H. Djingarey, N. Messonnier, T. A. Clark, J. L. Kambou, R. Novak, F. V. K. Diomandé, I. Medah, M. L. Jackson, Identifying optimal vaccination strategies for serogroup A *Neisseria meningitidis* conjugate vaccine in the African meningitis belt, PLoS One 8 (5) (2013) e63605.

[33] B. Lopman, K. Simmons, M. Gambhir, J. Vinjé, U. Parashar, Epidemiologic implications of asymptomatic reinfection: a mathematical modeling study of norovirus, Am J Epidemiol 179 (4) (2014) 507–12.

[34] V. E. Pitzer, C. C. Bowles, S. Baker, G. Kang, V. Balaji, J. J. Farrar, B. T. Grenfell, Predicting the impact of vaccination on the transmission dynamics of typhoid in South Asia: a mathematical modeling study, PLoS Negl Trop Dis 8 (1) (2014) e2642.

[35] F. Weidemann, M. Dehnert, J. Koch, O. Wichmann, M. Höhle, Bayesian parameter inference for dynamic infectious disease modelling: rotavirus in Germany, Stat Med 33 (9) (2014) 1580–99.

[36] D. G. Regan, D. P. Wilson, J. S. Hocking, Coverage is the key for effective screening of *Chlamydia trachomatis* in Australia, J Infect Dis 198 (3) (2008) 349–58.

[37] T. N. Doan, D. C. M. Kong, C. Marshall, C. M. J. Kirkpatrick, E. S. McBryde, Modeling the impact of interventions against *Acinetobacter baumannii* transmission in intensive care units, Virulence 7 (2) (2016) 141–52.

[38] C. Hogea, T. Van Effelterre, A. Cassidy, A model-based analysis: what potential could there be for a *S. aureus* vaccine in a hospital setting on top of other preventative measures?, BMC Infect Dis 14 (2014) 291.

[39] X. Wang, S. Panchanathan, G. Chowell, A data-driven mathematical model of CA-MRSA transmission among age groups: evaluating the effect of control interventions, PLoS Comput Biol 9 (11) (2013) e1003328.

[40] G. F. Webb, M. A. Horn, E. M. C. D'Agata, R. C. Moellering, Jr, S. Ruan, Competition of hospital-acquired and community-acquired methicillin-resistant *Staphylococcus aureus* strains in hospitals, J Biol Dyn 4 (1) (2010) 115–29.

[41] C. M. Macal, M. J. North, N. Collier, V. M. Dukic, D. T. Wegener, M. Z. David, R. S. Daum, P. Schumm, J. A. Evans, J. R. Wilder, L. G. Miller, S. J. Eells, D. S. Lauderdale, Modeling the transmission of community-associated methicillin-resistant *Staphylococcus aureus*: a dynamic agent-based simulation, J Transl Med 12 (2014) 124.

[42] F. Chamchod, S. Ruan, Modeling methicillin-resistant *Staphylococcus aureus* in hospitals: transmission dynamics, antibiotic usage and its history, Theor Biol Med Model 9 (2012) 25.

[43] S. R. Deeny, C. J. Worby, O. Tosas Auguet, B. S. Cooper, J. Edgeworth, B. Cookson, J. V. Robotham, Impact of mupirocin resistance on the transmission and control of healthcare-associated MRSA, J Antimicrob Chemother 70 (12) (2015) 3366–78.

[44] R. Zadoks, H. Allore, T. Hagenaars, H. Barkema, Y. Schukken, A mathematical model of *Staphylococcus aureus* control in dairy herds, Epidemiol Infect 129 (02) (2002) 397–416.
